# Bioactive Glass Modified with Zirconium Incorporation for Dental Implant Applications: Fabrication, Structural, Electrical, and Biological Analysis

**DOI:** 10.3390/ijms241310571

**Published:** 2023-06-24

**Authors:** Imen Hammami, Sílvia Rodrigues Gavinho, Ana Sofia Pádua, Isabel Sá-Nogueira, Jorge Carvalho Silva, João Paulo Borges, Manuel Almeida Valente, Manuel Pedro Fernandes Graça

**Affiliations:** 1 I3N and Physics Department, Aveiro University, 3810-193 Aveiro, Portugal; imenhammami@ua.pt (I.H.); silviagavinho@ua.pt (S.R.G.); mav@ua.pt (M.A.V.); 2 I3N-CENIMAT and Physics Department, NOVA School of Science and Technology, Campus de Caparica, 2829-516 Caparica, Portugal; as.padua@campus.fct.unl.pt (A.S.P.); jcs@fct.unl.pt (J.C.S.); 3 Associate Laboratory i4HB—Institute for Health and Bioeconomy, NOVA School of Science and Technology, NOVA University Lisbon, 2819-516 Caparica, Portugal; isn@fct.unl.pt; 4 UCIBIO—Applied Molecular Biosciences Unit, Department of Life Sciences, NOVA School of Science and Technology, NOVA University Lisbon, 2819-516 Caparica, Portugal; 5 I3N-CENIMAT and Materials Science Department, NOVA School of Science and Technology, Campus de Caparica, 2829-516 Caparica, Portugal; jpb@fct.unl.pt

**Keywords:** Bioglass^®^, zirconium, dielectric properties, osseointegration, antibacterial properties, bioactivity, implant coating

## Abstract

Implantology is crucial for restoring aesthetics and masticatory function in oral rehabilitation. Despite its advantages, certain issues, such as bacterial infection, may still arise that hinder osseointegration and result in implant rejection. This work aims to address these challenges by developing a biomaterial for dental implant coating based on 45S5 Bioglass^®^ modified by zirconium insertion. The structural characterization of the glasses, by XRD, showed that the introduction of zirconium in the Bioglass network at a concentration higher than 2 mol% promotes phase separation, with crystal phase formation. Impedance spectroscopy was used, in the frequency range of 10^2^–10^6^ Hz and the temperature range of 200–400 K, to investigate the electrical properties of these Bioglasses, due to their ability to store electrical charges and therefore enhance the osseointegration capacity. The electrical study showed that the presence of crystal phases, in the glass ceramic with 8 mol% of zirconium, led to a significant increase in conductivity. In terms of biological properties, the Bioglasses exhibited an antibacterial effect against *Gram-positive* and *Gram-negative* bacteria and did not show cytotoxicity for the Saos-2 cell line at extract concentrations up to 25 mg/mL. Furthermore, the results of the bioactivity test revealed that within 24 h, a CaP-rich layer began to form on the surface of all the samples. According to our results, the incorporation of 2 mol% of ZrO_2_ into the Bioglass significantly improves its potential as a coating material for dental implants, enhancing both its antibacterial and osteointegration properties.

## 1. Introduction

The loss of teeth is considered a major problem that affects millions of patients, and it can negatively impact their social life, self-confidence, and well-being. According to the World Health Organization (WHO), globally, an estimated 7% of people aged 20 years or older are affected by tooth loss, while a prevalence of 23% for people aged 60 years or older is estimated [1]. While removable prostheses have been an effective solution for decades, the implant therapeutic solution has become now a widespread and credible alternative to respond to the loss of natural teeth. Despite its advantages such as the preservation of adjacent teeth and long-term success, implant failure can still occur. The formation of biofilms surrounding implants is frequently observed after implantation surgeries. The biofilm is a thin layer of microorganisms of different species that adhere to a surface and secrete a sticky, protective matrix known as an extracellular polymeric substance (EPS). When bacteria colonize the surface of an implant and develop a biofilm, this can result in infection and inflammation in the surrounding tissue. This may develop in peri-implantitis, a chronic inflammatory disease that gradually damages the bone around the implant. Currently, no therapy can ensure the total removal of bacterial biofilm. However, the use of multifunctional coatings that increase the implant’s ability to integrate with the surrounding bone and at the same time prevent bacterial colonization can reduce the risk of peri-implant disease.

The 45S5 Bioglass^®^ (46.1 SiO_2_, 24.4 Na_2_O, 26.9 CaO, 2.6 P_2_O_5_, mol%) proposed by Hench et al. is successful as a coating for implants [2,3]. This Bioglass can form the hydroxyapatite layer on its surface that promotes the growth of new bone and improves the stability of the implant by integrating it with the surrounding tissue [3,4,5,6]. Additionally, 45S5 Bioglass has antimicrobial properties against a range of microorganisms, including bacteria and fungi [7,8,9]. This is due to the release of ions such as sodium and calcium from the Bioglass, which can disrupt the cell membrane and inhibit the growth of microorganisms.

Several studies have reported that the insertion of inorganic ions such as iron, copper, silver, niobium, zirconium, etc., into the glass network improves its biological response [10,11,12,13,14,15]. The use of zirconium (Zr) has been actively studied due to its superior mechanical properties, biocompatibility, and excellent osseointegration property, making it a promising material for use in regenerative medicine [16,17,18]. Zirconium has been found to promote the proliferation of osteoblast cells without showing any detrimental reactions [16,19,20]. In addition, it has been shown to stimulate osteoinduction in in vitro and in vivo evaluations [21]. Moreover, it has been ascertained that zirconium oxide (ZrO_2_) particles exhibit remarkable antimicrobial properties in relation to Gram-positive and Gram-negative bacterial strains including *Pseudomonas aeruginosa*, *Escherichia coli*, *Staphylococcus aureus*, *Staphylococcus epidermidis*, and *Bacillus subtilis* [22,23,24].

The objective of the current study is to address a prominent issue in the dental implant field, specifically bacterial infection, which often leads to bone support loss and subsequent rejection of the implant. Our aim is to tackle these challenges by developing a multifunctional material for implant coating that exhibits superior antibacterial function and rapid osteointegration. For this purpose, Bioglass 45S5 modified by the insertion of different percentages of zirconium was synthesized by the melt-quenching method. Morphological, structural, and biological investigations were carried out on these Bioglasses in order to evaluate the potential of using this material as a coating for dental implants. Furthermore, the electrical properties of the Bioglass samples were also examined due to their potential to improve the osteoconductive and osseointegration capacity of implants through electrical polarization.

## 2. Results and Discussion

### 2.1. Structural Characterization

**Figure 1:**
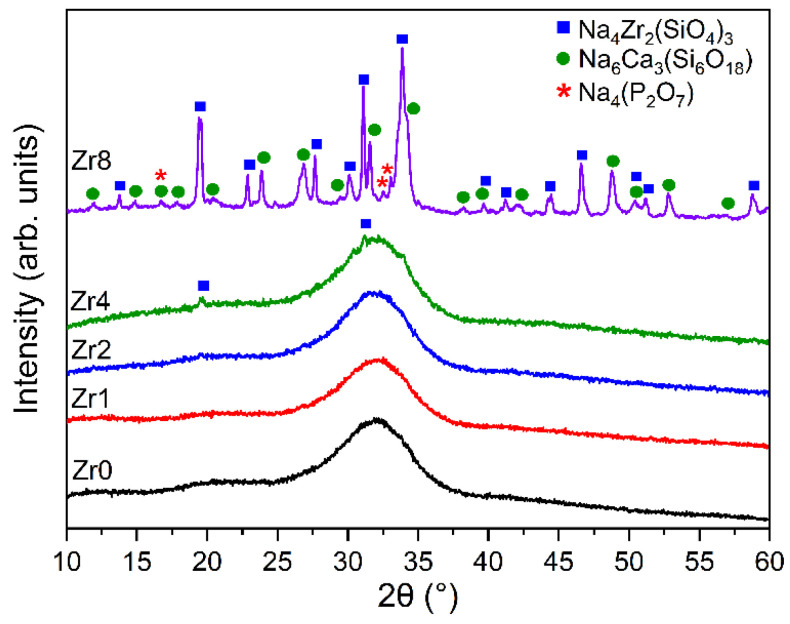
XRD patterns of 45S5 Bioglass samples modified by the insertion of various amounts of ZrO_2_.

The XRD patterns of the Bioglass samples modified by the insertion of ZrO_2_ are shown in Figure 1. The 45S5 Bioglass sample, Zr0, did not reveal any sharp or discrete diffraction peaks but instead shows broad bands characteristic of amorphous materials [11,15,25]. The insertion of a small quantity of ZrO_2_ (less than 2 mol%) into the glass network did not change the amorphous characteristics of the 45S5 Bioglass. However, by increasing the concentration of ZrO_2_, new crystalline phases were observed. This behavior has already occurred in Bioglass containing zirconium [23]. For the Bioglass containing 8 mol% of ZrO_2_, crystalline phases were identified, such as sodium zirconium silicate (Na_4_Zr_2_(SiO_4_)_3_) with a hexagonal crystalline structure, sodium calcium silicon oxide (Na_6_Ca_3_(Si_6_O_18_)) with a hexagonal crystal system, and sodium phosphate (Na_4_(P_2_O_7_)) that exhibits an orthorhombic structure. Moreover, the XRD pattern of the Bioglass modified with the insertion of 4 mol% of ZrO_2_ shows the presence of (Na_4_Zr_2_(SiO_4_)_3_). From the XRD patterns, it is possible to conclude that adding a high amount of ZrO_2_ to the 45S5 Bioglass leads to the formation of crystal phases. As a result of this crystal formation, the glass is no longer in the glass-forming region of this system.

**Figure 2:**
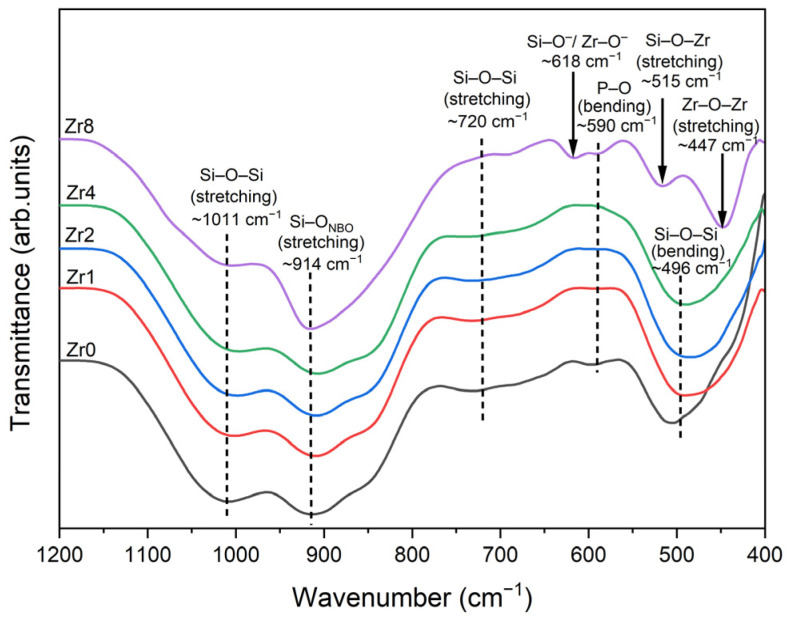
FTIR spectra of 45S5 Bioglass samples modified by ZrO_2_ insertion.

In Figure 2, the FTIR spectra of the glasses illustrate various features. The bands detected at approximately 1011 cm^−1^ and 720 cm^−1^ are linked to the Si–O–Si stretching modes. A band at 914 cm^−1^ was observed and attributed to the Si–O_NBO_ stretching mode, indicating the presence of non-bridging oxygen (NBO) ions. A noticeable intensification of this band was observed for the Zr8 sample as compared to the spectra of the other glass samples, thus indicating an increase in the number of NBO ions linked to silicon Si atoms within the glass network. This increase in NBO concentration is likely attributed to the formation of the Na_6_Ca_3_(Si_6_O_18_) crystal phase as shown in the XRD analysis. Additionally, a shoulder observed at 590 cm^−1^ is associated with the bending vibration of the P–O molecule. The Si–O–Si bending mode is linked to the band appearing at around 496 cm^−1^ [15,26,27,28,29,30,31].

Similar to the XRD results, changes in the phases were observed in the FTIR spectra of the sample with a high concentration of ZrO_2_. Additional bands were observed for the sample Zr8, at 447 cm^−1^ and 515 cm^−1^ attributed to Zr–O–Zr asymmetric stretching vibrations and Zr–O–Si stretching vibrations, respectively [32,33,34,35]. Moreover, a band was identified at 618 cm^−1^, although its assignment is unclear. According to the literature, this band could be assigned to the vibration of Si–O^−^ groups or Zr–O^−^ vibrations [35,36].

### 2.2. Thermal Analysis

**Figure 3:**
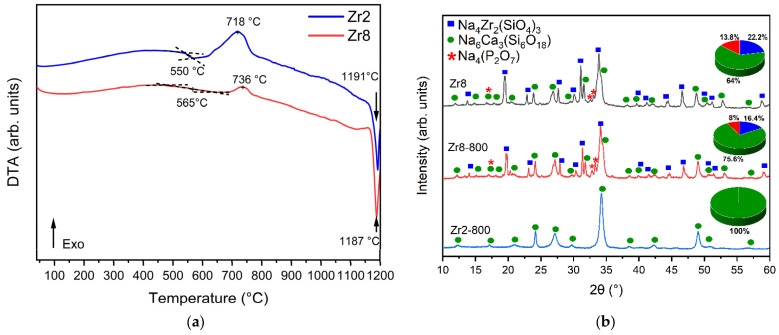
(**a**) DTA spectrum of the Zr2 and Zr8 samples and (**b**) XRD patterns of Zr2 and Zr8 after thermal treatment at 800 °C for 6 h.

Figure 3a presents the differential thermal analysis (DTA) thermograms of the Bioglasses with 2% and 8% ZrO_2_. The results show that the increase in the ZrO_2_ content from 2 to 8 mol% induces a rise in the glass transition temperature T_g_ from 550 °C to 565 °C, thus indicating the improvement in the rigidity of the glass due to the presence of the crystalline phases as shown by XRD. Table 1 shows the characteristic temperature of the glasses modified with ZrO_2_. Compared to the Bioglass base, the addition of 2 mol% of ZrO_2_ results in a decrease in both the glass transition temperature (T_g_) and the crystallization temperature (T_c_). This reduction can be attributed to an increase in the number of non-bridging oxygens (NBOs) present within the glass network. Conversely, the insertion of 8 mol% of ZrO_2_ in the glass network leads to an increase in the characteristic temperature. This increase can be attributed to a decrease in the number of non-bridging oxygens (NBOs) within the glass network due to crystal formation. The reduction in such NBOs will promote a more rigid global network, resulting in a higher characteristic temperature.

Moreover, the exothermic peak associated with the formation of crystalline phases is larger and shifted to a higher temperature for the sample Zr2 compared to the Zr8 sample. To examine this change in the exothermic peak, an XRD analysis was performed on the Zr2 and Zr8 samples thermally treated at 800 °C for 6 h (designated as Zr2-800 and Zr8-800). According to the XRD results presented in Figure 3b, the exothermic peak observed for the Zr2 sample is attributed to the formation of the Na_6_Ca_3_(Si_6_O_18_) phase. The decrease in the crystallization peak for the Zr8 sample is related to the growth of the Na_6_Ca_3_(Si_6_O_18_) phase as a result of the relative decrease in Na_4_Zr_2_(SiO_4_)_3_ and Na_4_(P_2_O_7_) phases.

### 2.3. Electrical Characterization

**Figure 4:**
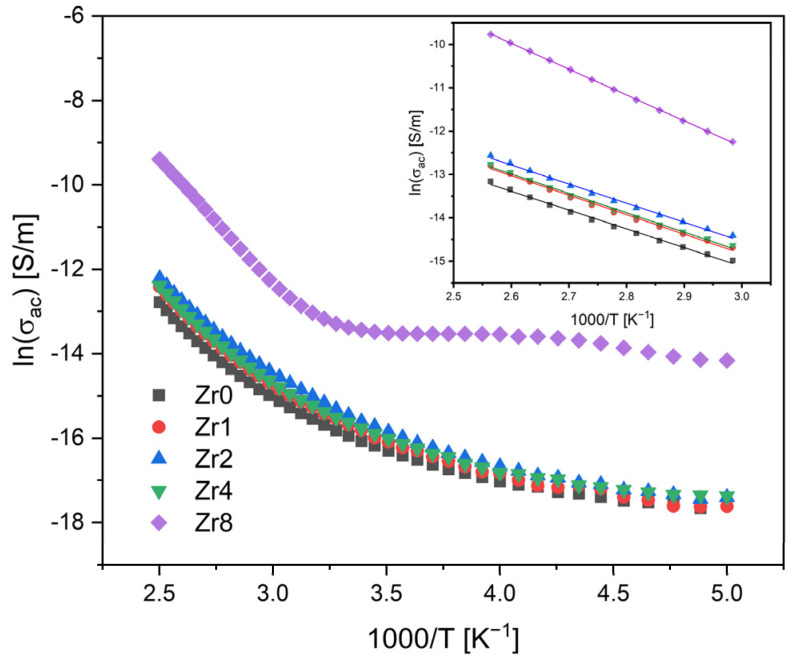
AC conductivity versus 1000/T at 10 kHz (inset: magnification of the high-temperature measurement zone; the lines reflect the Arrhenius fit).

Figure 4 shows the AC conductivity versus 1000/T on a semi-logarithmic scale. In general, two main mechanisms can contribute to the conductivity of glasses: ionic and electronic. In the case of glass ceramics, the presence of crystalline phases and phase boundaries can significantly affect conductivity [37,38]. The concentration and distribution of these crystalline phases can alter the local electric field and influence the motion of the charge carriers. This is because the conductivity will be different depending on whether the charge carriers are in a glass or crystalline environment.

The results obtained show that the AC conductivity tends to increase with the temperature, due to the increased mobility of the charge carriers. In high-temperature regions, the variation in the conductivity becomes linear, suggesting that the Arrhenius model can be utilized to determine the activation energy. The calculated activation energy for all samples and the AC and DC conductivity values are compiled in Table 2.

The results indicate that samples modified with Zr concentrations ranging from 0 to 4 mol% demonstrate similar values of AC activation energy and conductivity. However, the Zr8 sample displays higher values, suggesting that the process of conduction in this sample is controlled by different mechanisms. The conductivity in the bioactive glass system is predominantly linked to the energy carried by network modifier ions, Na^+^ and Ca^2+^, moving through the glass network [15,39]. However, the transition of the Bioglass structure from amorphous to crystalline can considerably impede the mobility of these ions by trapping them within the crystal phases. Therefore, in crystalline Bioglasses, such as the Zr8 sample, the principal contributors to the conduction mechanism are the crystal phases dispersed within the glass matrix.

**Figure 5:**
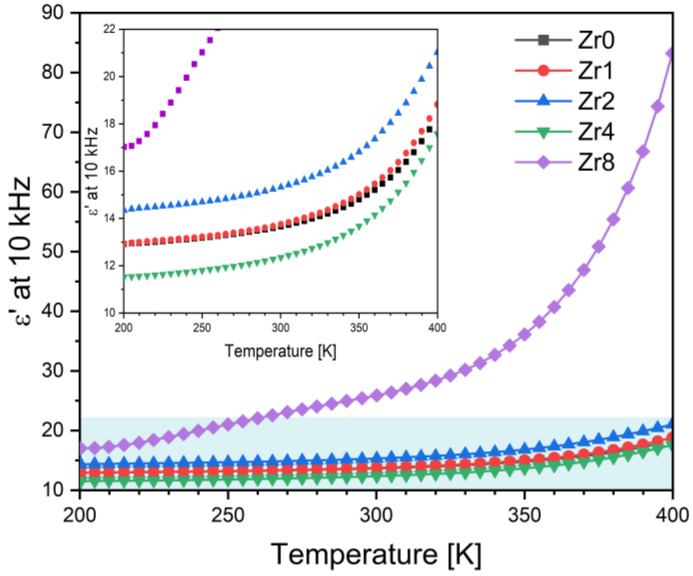
The dielectric constant, ε’, as a function of the temperature of the Bioglass samples modified by ZrO_2_ insertion at 10 kHz (the inset is the magnification of the blue rectangle region).

Figure 5 illustrates the temperature dependence of the dielectric constant, ε’, of all the Bioglasses measured at a frequency of 10 kHz. The results demonstrate that the dielectric constant increases with temperature for all samples. Furthermore, it has been observed that the addition of ZrO_2_ to a concentration of 2 mol% increases the dielectric constant of the Bioglass. However, further increases in the concentration of ZrO_2_ to 4 mol% result in a decrease in the dielectric constant. On the other hand, the addition of 8 mol% of ZrO_2_ significantly increases the value of the dielectric constant in comparison to the other samples. This trend is evident from the data in Table 2, which reports the values at room temperature. The decrease in the dielectric constant in the Zr4 glass could be related to the presence of the Na_4_Zr_2_(SiO_4_)_3_ crystal phase. The observed high values of the dielectric constant for the Zr8 glass could be attributed to the presence of Na_6_Ca_3_(Si_6_O_18_) and Na_4_(P_2_O_7_) crystal phases which accounts for approximately 64% and 13.8% of the crystal phase, respectively, as shown in Figure 3b.

**Figure 6:**
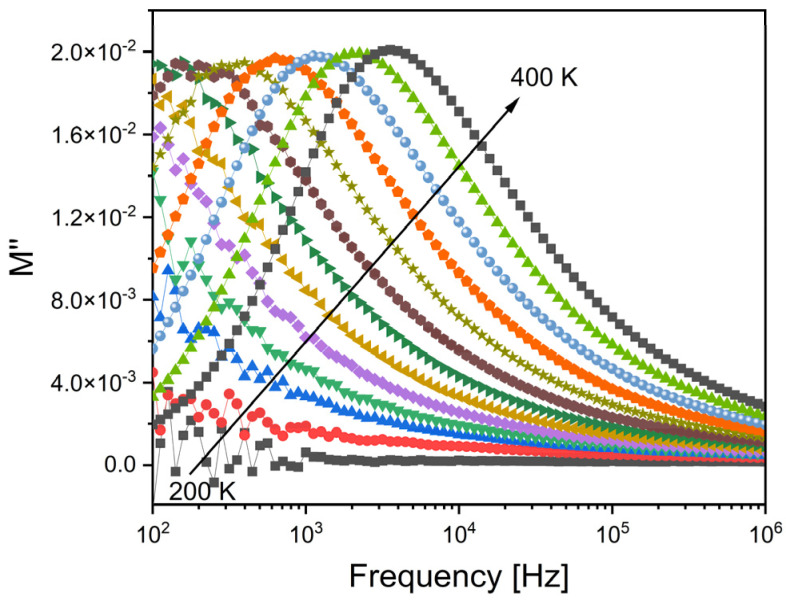
The imaginary part of the dielectric modulus, M″, versus the frequency for the Zr2 sample.

The analysis of the dielectric properties of the samples was then conducted using the modulus formalism, M*, which is defined as 1/ε*, and gives the advantage of mitigating the influence on the dielectric data from low capacitance contributions, like electrode polarization and also low-frequency conductivity [40]. The obtained results, as depicted in Figure 6, indicate the presence of a single dielectric relaxation process that shifts towards higher frequencies as the temperature increases. Notably, alternative approaches like permittivity or impedance or admittance did not reveal the presence of this dielectric relaxation behavior. Hence, it can be inferred that the relaxation phenomenon observed is associated with an inherent characteristic linked to the formation of dipoles between the network modifier ions and the non-bridging oxygen ions present structurally in their vicinity.

**Figure 7:**
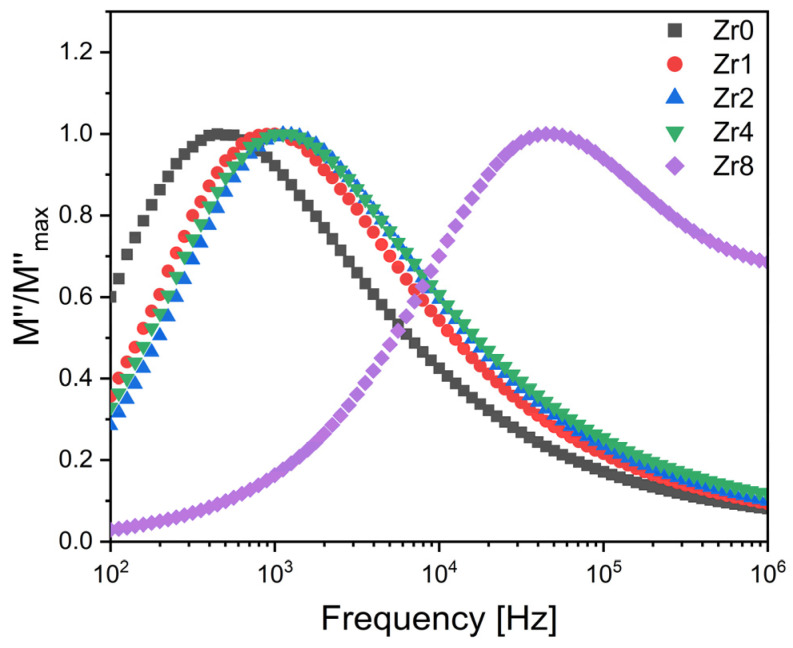
The normalized imaginary part of the modulus M″/M″max versus the frequency at 380 K for the Bioglass samples modified by ZrO_2_ insertion.

A comparison of normalized imaginary parts of the electric modulus M″/M″_max_ as a function of frequency at different concentrations of ZrO_2_ inserted into the Bioglass network is presented in Figure 7. The results obtained show a slight shift in the electrical modulus relation peak to a higher frequency when increasing the concentration of zirconium from 0 to 4 mol%, suggesting a decrease in the relaxation time and thus an increase in the charged species and dipoles from the glass matrix. Furthermore, it can be observed that increasing the zirconium concentration to 8 mol% leads to a significant shift in dielectric relaxation towards the high-frequency range. This significant shift could be due to the alteration of the mechanisms responsible for this relaxation phenomenon. According to the literature, we propose that this relaxation can be attributed to the dipoles present in both the crystallites and the crystal–glass interfaces [25].

### 2.4. Cytotoxicity Assay

**Figure 8:**
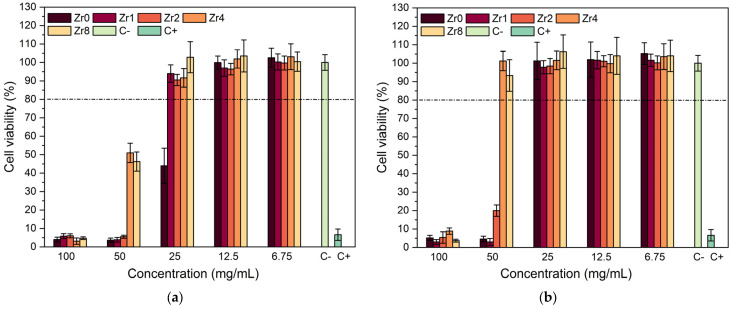
Relative viability of (**a**) non-passivated and (**b**) passivated Bioglass extracts modified by ZrO_2_ in culture with Saos-2 cells. C+ and C− represent the positive control (cells in a cytotoxic environment) and negative control (viable cells).

Figure 8 presents the Saos-2 cell line viability in response to exposure to Bioglass extracts. This study aimed to assess the biocompatibility of the compositions examined for application in bone regeneration. The effect of extract contact with the cell line was determined using a resazurin assay to evaluate cell viability. Our findings reveal that extracts that were not preconditioned with McCoy’s culture medium (non-passivated extracts) exhibited considerable cytotoxicity, resulting in cell viability below 10% at concentrations of 100 mg/mL. At a concentration of 50 mg/mL, the Zr4 and Zr8 samples showed an improvement in cell viability, although the cytotoxic behavior remained evident. However, a dilution of the extract to 25 mg/mL decreased the cytotoxicity, and the samples containing zirconium demonstrated higher cell viability compared to the Bioglass base sample. This suggests that the incorporation of ZrO_2_ into the bioactive glass can enhance the biocompatibility of the materials, corroborating previous research studies [10,41,42]. The cytotoxicity of the extracts can be mitigated by subjecting the materials to a passivation process, as evidenced in Figure 8b. It is noteworthy that cytotoxicity is associated with an elevation in local pH due to a surge in ion-exchange reactions upon contact with the cell culture medium within the first 24 h [29]. Upon contact with the cellular medium, bioactive glass undergoes a breakdown of its Si-O-Si bonds, resulting in the release of soluble silica in the form of Si(OH)_4_ into the solution which increases the dissolution rate and pH of the surrounding environment, thus affecting cellular metabolism and function. When the samples are passivated, the impact of pH alkalinization induced by bioactive glasses decreases.

The passivated extracts from the Zr4 and Zr8 samples were found to be non-cytotoxic, even at a high concentration of 50 mg/mL. Similarly, the Bioglass base and the Bioglasses with low concentrations of ZrO_2_ also did not show any cytotoxicity at an extract concentration of 25 mg/mL. These results suggest that, at these concentration levels, the samples no longer pose a threat to the organism as they are able to be naturally regulated by in vivo pH regulation processes [43,44].

### 2.5. Antibacterial Activity

**Figure 9:**
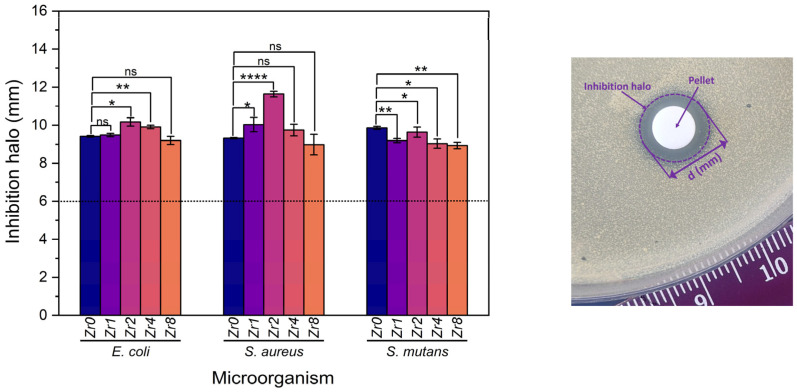
Measurements of inhibition halo diameter of the Bioglass samples against *E. coli*, *S. aureus*, and *S. mutans* bacteria after incubation for 24 h. The results are reported as the mean ± SD. The asterisks indicate significance in an unpaired *t*-test; * *p* ≤ 0.05; ** *p* ≤ 0.01; **** *p* ≤ 0.0001, ns: nonsignificant. The image on the right side is an example of an assay plate illustrating the inhibition halo of the Zr2 pellet on *S. aureus*.

Ensuring the prevention of implant-related biofilm has emerged as a critical aspect of the success of implantation therapy. Even though 45S5 bioactive glass has demonstrated exceptional osteogenic and antibacterial properties [7,9,45], it is important to investigate the antibacterial potential of Bioglass modified with zirconium. Figure 9 shows the results obtained from the evaluation of the antibacterial properties of the produced Bioglasses using the agar disc diffusion method. The results confirm that all samples exhibit antibacterial activity against all bacterial strains as an inhibition zone was observed around the Bioglass pellets with mean values higher than 6 mm, the diameter of the pellets. Changes in pH to the alkaline range and osmotic pressure, induced by the dissolution of Bioglass ions such as Na^+^ and Ca^2+^ into the medium, are two mechanisms associated with the antibacterial effect of 45S5 Bioglass against certain oral bacteria [7,46]. An alkaline pH range creates an unfavorable environment for bacterial growth and metabolism, causing a morphological change in the bacteria and their death. The release of ions and changes in their concentration in the bacterial environment affects the bacteria cell membrane causing a reduction in pressure across it that modifies the cell size, shape, and membrane tension levels. Moreover, other studies have revealed that ZrO_2_ exhibits a potential inhibitory effect on a certain bacterium [22,23,47]. This effect is caused by the positive charge transmitted by ZrO_2_ particles that could interact with negatively charged bacterial cells through electromagnetic forces. These attractions can potentially cause the bacterial cell wall to rupture, leading to cell death [22,48]. This could explain the increase in the antibacterial activity of the Bioglass modified by ZrO_2_ compared to the Bioglass base.

The sample modified by the insertion of 2 mol% of Zr, Zr2, shows the highest antimicrobial effect compared to the other Bioglass samples modified with Zr with the means of the inhibition halo of 10.17 mm, 11.63 mm, and 9.63 mm against *E. coli*, *S. aureus*, and *S. mutans* bacteria, respectively. Upon exceeding the Zr concentration to 2 mol%, a discernible reduction in the inhibition halo was observed against *S. aureus* and *E. coli*, indicative of a concomitant decrease in the antibacterial efficacy of the Bioglass. This decrease is attributed to a structural alteration of the Bioglass from amorphous to crystalline with increasing zirconium concentration, as depicted in Figure 1. Indeed, the Bioglass structure can affect its dissolution rate and ion release. In general, amorphous Bioglass exhibits a higher dissolution rate compared to crystalline Bioglass [49]. This can be attributed to the fact that ions present in the glass network, such as Na^+^ and Ca^2+^, tend to be entrapped within the crystalline phases of the Bioglass, which consequently decreases its dissolution rate.

### 2.6. In Vitro Bioactivity Assay

**Figure 10:**
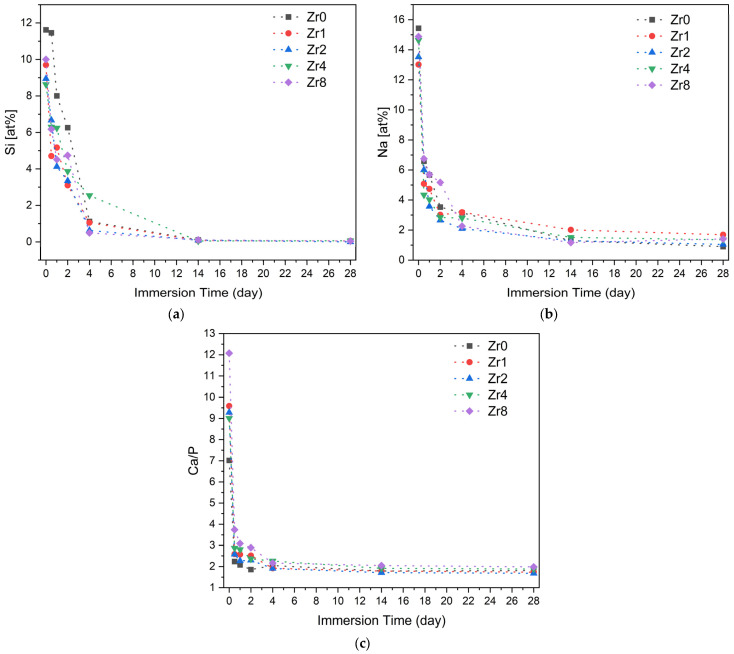
The variation in the concentration of (**a**) Si and (**b**) Na and (**c**) the Ca/P ratio on the Bioglass pellets’ surfaces after soaking in SB F.

To assess the ability of the bioactive glasses to promote new bone formation and integration with the host bone, an in vitro test was conducted to evaluate the formation of an apatite layer when the material is immersed in simulated body fluid (SBF). This method provides insight into the physicochemical reactions occurring at the surface of the Bioglass in a biological medium. The formation of an apatitic layer is critical for osteoblast cell adhesion and proliferation [50]. Figure 10 shows the variation in the Si and Na atomic percentages and the Ca/P ratio, evaluated by EDS analysis on the surface of the Bioglass pellets after immersion in SBF for various time intervals. Upon contact with SBF, the bioactive glass undergoes a reaction mechanism involving an ionic exchange between the sample and its surrounding medium. First, the alkali and alkaline earth ions (Na^+^ and Ca^2+^) on the glass surface are exchanged with H^+^ and H_3_O^+^ ions in the medium, resulting in an increase in pH. This increase in pH values facilitates the breakdown of Si-O-Si bonds in the glass network, which accelerates the dissolution of the glass and leads to the formation of silanol units (Si(OH)_4_). These silanol units condense on the glass surface to form a hydrated silica layer, which acts as a nucleation site for the growth of a carbonated hydroxyapatite layer which will subsequently crystallize [51,52].

According to the EDS results shown in Figure 10, the fast drop in the Si and Na concentration on the Bioglass surface in the first days of SBF immersion is attributed to the dissolution of these elements into the medium and the formation of an amorphous calcium phosphate layer. Prolonged Bioglass soaking in SBF leads to stabilization of ion concentration and a decline in the Ca/P ratio, reaching a value close to that of hydroxyapatite in the natural bone (Ca/P ≈ 1.67) [53,54], thus indicating the development of an apatite layer.

**Figure 11:**
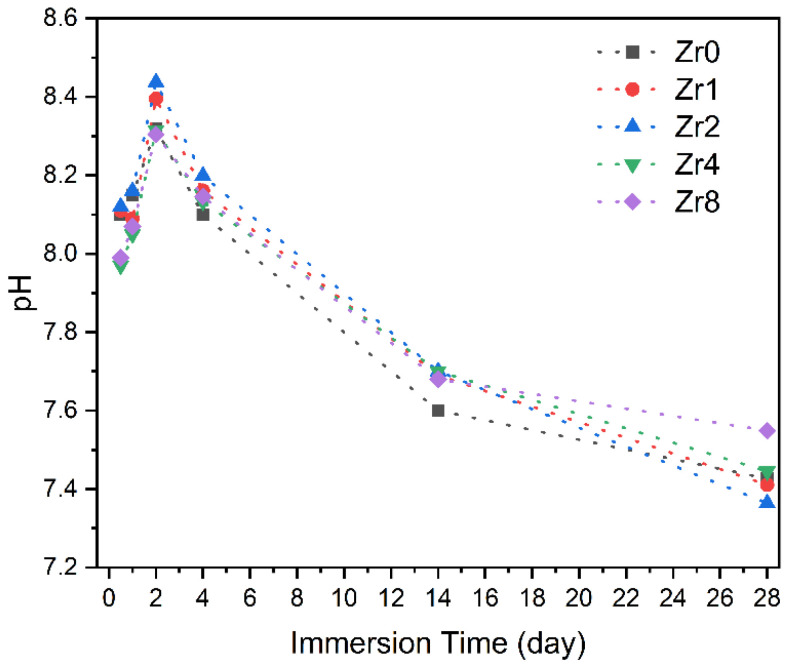
The variation in the pH of the SBF solution with the immersion time.

Figure 11 presents the pH variation of the SBF medium after Bioglass pellets’ immersion for different time intervals, ranging from 12 h to 28 d. It is worth noting that during the immersion of the Bioglass in SBF, the medium was replaced every two days to mimic the continuous flow of biological fluids that would be present when the Bioglass is implanted in an organism. In the first 2 days, the pH of the SBF medium after immersion of the glasses exhibited an increase when compared to the initial pH of the SBF solution (7.4). This increase is attributed to the dissolution of the alkaline metal ions (Na^+^ and Ca^2+^) from the Bioglass samples. After 2 days, the pH value of the SBF medium tends to decrease with the immersion time which suggests the creation of the apatite layer on the surface of the bioactive glass.

**Figure 12:**
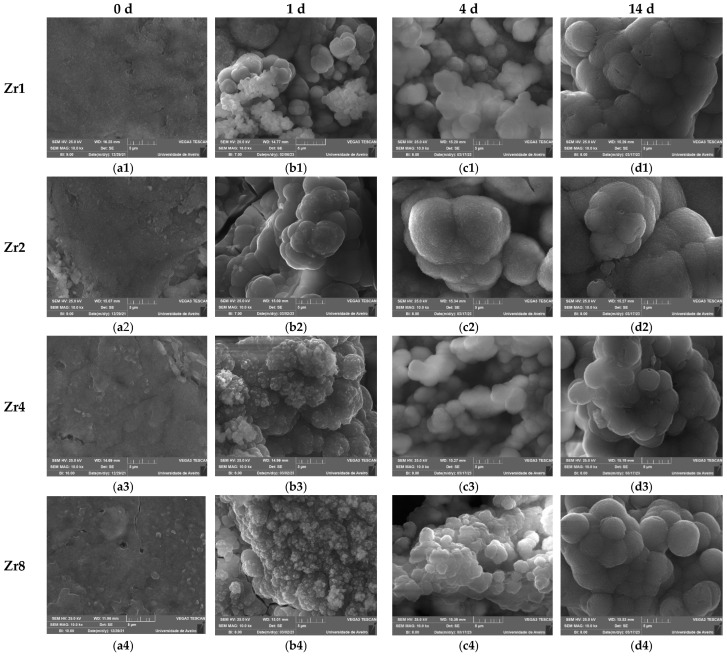
SEM micrographs of the Bioglasses containing various concentrations of ZrO_2_ after immersion in SBF for: (**a1**–**a4**) 0 d; (**b1**–**b4**) 1 d; (**c1**–**c4**) 4 d; (**d1**–**d4**) 14 d. (The magnification of the SEM images is 10 kX).

The formation of this calcium-phosphate-rich layer was also determined using SEM observation. Figure 12 shows the SEM micrographs taken at the surface of the Bioglass pellets after immersion in SBF for 0 d, 1 d, 4 d, and 14 d. The bioactivity of the glasses was confirmed through SEM analysis, which revealed the presence of spherical (cauliflower-like) particles indicative of apatitic layer formation on the surface of the samples. As the immersion time in SBF increased, the apatite particles agglomerate and become denser, eventually resulting in a fully coated surface with an apatite layer at 14 d of immersion. These observations demonstrate that the prepared glasses exhibit significant bioactive properties, suggesting their potential use in bone regeneration. Moreover, the comparison of samples with different ZrO_2_ content revealed that the insertion of 2 mol% of ZrO_2_ promoted the bioactivity of the Bioglass within the first 24 h, as this sample exhibited larger spheroidal apatite particles, indicative of an optimal composition for apatite formation. However, beyond this concentration, the apatite particles became smaller during the first days of SBF immersion. This phenomenon could be attributed to changes in the crystalline structure of the sample with the insertion of more than 2 mol% of ZrO_2_, resulting in reduced dissolution and ion exchange of the Bioglass and, hence, reduced bioactivity. These findings suggest that the optimal ZrO_2_ concentration for promoting the bioactivity of the glasses is around 2 mol%.

## 3. Materials and Methods

### 3.1. Sample Preparation

The 45S5 Bioglass^®^ (46.1 SiO_2_, 24.4 Na_2_O, 26.9 CaO, and 2.6 P_2_O_5_, mol%) containing several percentages of zirconium dioxide (ZrO_2_), from 0 to 8 mol% (designed by Zr0, Zr1…Zr8) was synthesized using the melt-quenching method. The chemical precursors SiO_2_, P_2_O_5_, CaCO_3_, Na_2_CO_3_, and (ZrO(NO_3_)_2_∙XH_2_O; X~3) with high purity grade (>99.99%) were homogeneously mixed using a planetary ball milling process for 1 h at 300 rpm and the obtained powder was calcined at 800 °C for 8 h. The bulk glass samples were achieved by melting the calcined powder in platinum at 1300 °C for 1 h. To guarantee homogeneity during melting, the melt was frequently hand mixed. Subsequently, the bulk Bioglasses were well-ground and milled into fine powders using a planetary ball milling process for 1 h at 300 rpm.

### 3.2. Structural and Morphological Characterization

The X-ray diffractograms (XRD) were acquired at room temperature using a Malvern Panalytical Aeris powder diffractometer (CuK_α_ radiation, λ = 1.54056 Å). The acquisition was carried out using a scan step of 0.02° in 1 s in a 2θ angle range of 10–60°.

FT Perkin–Elmer Spectrum BX Spectrometer in the ATR crystal (Golden Gate Diamond ATR Accessory) was used to collect the FTIR spectra in the range of 400–1200 cm^−1^. Powder of each sample was used for the measurements. The room temperature and humidity were maintained at approximately 23 °C and 35% throughout the acquisition.

TESCAN Vega 3 scanning electron microscopy (SEM) was used to evaluate the samples surface morphology. The sample surfaces were coated with carbon before the microscopic observation to enhance the surface electron conductivity. A semiquantitative evaluation of the chemical composition of the samples was carried out using the Bruker EDS (energy dispersive spectroscopy) system in conjunction with the microscope. A 5 µm-diameter electron beam spot was used to analyse the surface of each sample at the sites.

### 3.3. Thermal Analysis

To examine the thermal characteristics of the glasses, differential thermal analysis (DTA) was conducted on a Hitachi STA 7300 apparatus with a controlled heating rate of 5 °C/min and under nitrogen N50 (99.999%) continuously flowing at a rate of 200 mL/min.

### 3.4. Electrical Characterization

The electrical properties of the bulk glass samples were characterized. The samples were carefully polished to produce parallel surfaces with a uniform thickness of approximately 1 mm. The silver conductive paste was then applied to each surface to form electrodes. Direct current conductivity (σ_DC_) measurements were conducted using a Keithley 617 electrometer, which is capable of detecting extremely low currents down to 10^−14^ A. During the measurement process, a voltage of 100 V was applied across the bulk glass over a temperature range of 200 to 400 K. The alternating current (σ_AC_) conductivity and impedance measurements were also measured over the same temperature range using an Agilent 4294 impedance meter, operating in the Cp–Rp configuration and with a broad frequency window from 100 Hz to 1 MHz. The temperature of the samples was controlled using an Oxford Research IT-C4 and monitored using a platinum sensor during both DC and AC measurements.

The complex electric permittivity ε* was calculated according to Equation (1) [55,56]:

ε* = ε’ − j ε’’ = Cp (d/ε_0_ A) − j d (ω Rp ε_0_ A), (1)

The activation energy was determined by adjusting the temperature dependence of the electrical conductivity using the Arrhenius model [38,57,58,59]:

σ = σ_0_ exp(−E_A_/(k_B_ T)), (2)

where σ_0_ is a pre-exponential factor, E_A_ is the activation energy, K_B_ is the Boltzmann constant, and T is the temperature.

### 3.5. Cytotoxicity Assay

The cytotoxicity of the samples was assessed using the extract method and human osteosarcoma cell line (Saos-2 cells, ATCC^®^ HTB-85™) in accordance with International Standard ISO 10993-5. Prior to the assessment, the Bioglass powders were sterilized for 2 h at 120 °C. To produce extracts, the samples were brought into contact with a culture medium (McCoy 5A medium, Merck KGaA, Darmstadt, Germany) at a concentration of 100 mg/mL. For the non-passivated extract, the medium was incubated with the powder for 24 h at 37 °C, filtered using a 0.22 µm cellulose acetate filter, and stored at 37 °C. For the passivated extract, fresh McCoy 5A medium was added to the same Bioglass powder and incubated for 24 h at 37 °C. The Saos-2 cells were seeded onto 96-well plates at a concentration of 30 k cells per cm^2^ and incubated for 24 h at 37 °C with a 5% CO_2_ atmosphere. Negative controls (C−, viable cells), positive controls (C+, cells in a cytotoxic environment where 10% of toxic compound dimethyl sulphoxide DMSO was added), non-passivated extracts, and passivated extracts, each with appropriate dilutions (50 mg/mL, 25 mg/mL, and 12.5 mg/mL), were added to the wells on the same plate and incubated for 48 h. The resazurin cell viability indicator was used to measure cell metabolism [60], and the optical absorbances of each well were measured at 570 nm and 600 nm using a Biotek ELX800 micro-plate reader. The study was performed in triplicate, with each experiment consisting of six replicates, to ensure the reproducibility of the results. 

### 3.6. Antibacterial Activity

The antibacterial activity of the Bioglasses containing several percentages of Zirconium was tested using the reference strains *Escherichia coli* K12 DSM498 (DSMZ, Braunschweig, Germany), *Staphylococcus aureus* COL MRSA (methicillin-resistant strain, supplied by Rockefeller University), and *Streptococcus mutans* DSM20523 (DSMZ, Braunschweig, Germany). The bacterial strains were cultured in tryptic soy broth (TSB) overnight at 37 °C. Before the test, pellets with a diameter of 6 mm and ~2 mm made of Bioglass powder were sterilized at 180 °C for 2 h.

The method of agar diffusion assay plates, using the two-layer bioassay, was performed with TSB medium solidified with agar 1.5% *w*/*v* (base layer) and 0.8% *w*/*v* (top layer). The plates were prepared with a base layer of 18–20 mL and 4 mL of molten seeded overlay containing about 10^8^ CFU/mL of the appropriate indicator bacteria. Pellets of the sample to be tested were placed in the center of the plates, left for 4 h at room temperature, and then incubated for 24 h at 37 °C. In the case of *S. mutans* the plates were kept in a 5% CO_2_ incubator.

Images of the pellets were taken, and the diameter of the inhibition halo was determined using ImageJ software; each pellet was measured 30 times in several orientations. GraphPad Prism 8.0 software was used to statistically analyze, with an unpaired *t*-test, the results of the three independent assays for each microorganism by comparing the bioactive glass base composition with each of the different samples.

### 3.7. In Vitro Bioactivity Assay

In accordance with ISO 23317:2017 Standards, the bioactivity assessment of the Bioglasses was carried out by immersing samples (pellets with a diameter of 7 mm consisting of powdered Bioglass) in simulated bodily fluid (SBF). The samples were put in various flasks, soaked in SBF, and placed in an incubator at 37 °C with continuous oscillating support for 12, 24, 48, 96 h, 14, and 28 d. To simulate the biological environment, the SBF solutions were renewed every 48 h.

To determine the volume of SBF needed for each sample, the following formula was applied:

V_s_ = 100 mm × S_a_, (3)

where V_s_ is the volume of SBF in mm^3^ and S_a_ is the surface area of the pellet in mm^2^.

After immersion, the pellets were removed from the SBF medium, gently cleaned with deionized water, and dried at room temperature. The samples were analyzed using SEM/EDS to determine the change in the ion concentration and the formation of an apatite-like layer on the surface over 28 days with the presence of a different percentage of zirconium.

## 4. Conclusions

Bioactive glasses containing zirconium were synthesized using the melt-quenching method. The structural characterization by XRD revealed that the addition of ZrO_2_ at a concentration above 2 mol% alters the Bioglass structure and promotes the formation of crystalline phases. This change from an amorphous to a crystalline structure had a significant impact on the biological properties of the sample, resulting in a decrease in both bioactivity and antibacterial effects. The Bioglass modified with 2 mol% of ZrO_2_ exhibited the most significant antibacterial effect against *E. coli*, *S. aureus*, and *S. mutans*. Hence, it can be inferred that from the prepared samples, the 45S5 bioactive glass modified with 2 mol% of ZrO_2_ is the most suitable composition for biomedical applications, including implant coating.

## Figures and Tables

**Table 1:** The characteristic temperatures for Zr0, Zr2, and Zr8.

Sample	T_g_ (°C)	T_c_ (°C)	T_m_ (°C)
Zr0 [25]	552	728	1175
Zr2	550	718	1191
Zr8	565	736	1187

**Table 2:** The dielectric constant (ε’), dielectric loss (tan δ), AC conductivity (σ_AC_), AC activation energy E_a_ (AC), DC conductivity (σ_DC_), and DC activation energy E_a_ (DC) for all Bioglass samples.

Sample	ε’	tan δ (×10^−2^)	σ_AC_ (×10^−6^) [S/m]	E_a_ (AC) [kJ/mol]	σ_DC_ (×10^−9^) [S/m]	E_a_ (AC) [kJ/mol]
	(300 K; 10 kHz)			(10 kHz)	(300 K)	
Zr0	13.59 ± 0.72	1.58 ± 0.02	11.92 ± 0.01	37.95 ± 0.98	0.91 ± 0.08	75.82 ± 0.79
Zr1	13.75 ± 1.92	2.02 ± 0.01	15.45 ± 0.07	39.09 ± 0.92	1.61 ± 0.16	73.20 ± 0.76
Zr2	15.32 ± 1.95	2.28 ± 0.03	19.46 ± 0.14	37.90 ± 0.78	1.19 ± 0.17	75.96 ± 0.79
Zr4	12.34 ± 1.53	2.37 ± 0.01	16.26 ± 0.09	38.68 ± 0.87	1.45 ± 0.19	73.20 ± 0.76
Zr8	25.88 ± 1.42	0.11 ± 0.02	154.99 ± 1.15	49.43 ± 0.15	33.2 ± 1.40	61.20 ± 0.63

## Data Availability

The data presented in this study are available from the corresponding author upon request.
